# 3D Laparoscopic common bile duct exploration with primary repair by absorbable barbed suture is safe and feasible

**Published:** 2021-07-16

**Authors:** Yen Pin Tan, Cheryl Lim, Sameer P Junnarkar, Cheong Wei Terence Huey, Vishalkumar G Shelat

**Affiliations:** ^1^Department of General Surgery, Tan Tock Seng Hospital, Singapore; ^2^Lee Kong Chian School of Medicine, Nanyang Technology University, Singapore

**Keywords:** 3D, common bile duct, endoscopy, laparoscopy, suture

## Abstract

**Background and aim:**

Endoscopic retrograde cholangiopancreatography (ERCP), with interval laparoscopic cholecystectomy (LC), is the most common treatment approach for common bile duct (CBD) stones. However, recent studies show that single-stage laparoscopic CBD exploration (LCBDE) is safe and feasible. Three-dimensional (3D) laparoscopy enhances depth perception and facilitates intracorporeal suturing. The application of 3D technology for LCBDE is emerging, and we report our case series of 3D LCBDE.

**Methods:**

We audited the 27 consecutive 3D LCBDE performed from July 2017 to January 2020. We have a liberal policy for magnetic resonance cholangiopancreatography (MRCP) in patients with deranged liver function tests (LFT). All CBD explorations were done through choledochotomy with a 5 mm flexible choledochoscope and primarily repaired with an absorbable barbed suture without a stent or T-tube.

**Results:**

The mean age of patients was 68 (range 44-91) years, and 12 (44%) were male. The indications for surgery were choledocholithiasis 67% (*n*=18), cholangitis 22% (*n*=6), and gallstone pancreatitis 11% (*n*=3). About 67% (*n*=18) had pre-operative ERCP. About 37% (*n*=10) had pre-operative biliary stent. Pre-operative MRCP was done in 74% (*n*=20), and the mean diameter of CBD was 14.5 mm (range 7–30). The median operative time was 160 (range 80–265) min. The operative drain was inserted in 18 patients. One patient each (4%) had a bile leak and a retained stone. There was no open conversion, readmission, or mortality.

**Conclusion:**

3D LCBDE with primary repair by an absorbable barbed suture is safe and feasible.

**Relevance for patients:**

This paper emphasized that one stage LCBDE should be a treatment option which is comparable with two stage ERCP followed by LC to treat CBD stones. In addition, 3D technology and barbed sutures use in LCBDE are safe and useful.

## 1. Introduction

Up to 10–15% of patients with gallstone disease have common bile duct stones (CBDS) [1]. Cystic duct stump blowout can occur if cholecystectomy is done before clearing CBD stones. Historically, open common bile duct exploration is the treatment of choice for CBDS. However, since the era of endoscopic retrograde cholangiopancreatography (ERCP), the standard treatment approach for CBDS is usually two-stage: ERCP with endoscopic stone clearance (first stage) followed by elective interval laparoscopic cholecystectomy (LC) (second stage). Laparoscopic common bile duct exploration (LCBDE) for the treatment of CBDS is another treatment option with comparable short- and long-term outcomes [2-6]. LCBDE offers the advantage of a single-stage procedure and averts potential morbidity from ERCP [7]. Besides, failure to cannulated, failure to extract stones, need for multiple ERCP sessions, and non-therapeutic ERCP due to spontaneously passed out stones are some inherent limitations of ERCP. LCBDE is not widely adopted due to the technical difficulty of intracorporeal suturing, training, and the need for special equipment (e.g. flexible choledochoscope). Koc *et al*. reported a randomized control trial of 120 patients comparing single-stage LCBDE and two-stage ERCP-LC and reported comparable outcomes [8].

Conversely, we have also reported that LCBDE can salvage a failed ERCP procedure [9]. We consider ERCP and LCBDE as complementary and not competing. The majority of published reports of LCBDE are with routine two-dimensional (2D) laparoscopy. Two-dimensional (2D) laparoscopy’s significant limitations include the lack of depth perception and spatial orientation loss with potential increasing surgical strain, risk of errors, and operative time [10]. 3D laparoscopy enhances depth perception, facilitates intracorporeal suturing and is increasingly reported to reduce laparoscopic surgery’s learning curve [11]. The adoption of LCBDE may be easier with the availability of 3D camera systems. Finally, many authors repair CBD over an internal stent or a T-tube, and primary CBD repair using absorbable barbed sutures is not common. We report a surgical audit of 3D LCBDE with primary closure of choledochotomy using absorbable barbed sutures.

## 2. Materials and Methods

### 2.1. Study population

We report an audit of electronic medical records of the first 27 consecutive patients treated by 3D LCBDE at our institution between July 2017 and January 2020. In our tertiary center, we performed an average of about 50 cases of major biliary procedures and 20–30 cases of LCBDE per year. 3D laparoscopy was introduced in 2017, and the patient pays an extra 3.5 USD equivalent for using the 3D laparoscopy. All 3D LCBDE cases were performed or supervised by one of the four consultant hepatobiliary surgeons in our unit. We have a liberal policy of magnetic resonance cholangiopancreatography (MRCP) for patients with suspected CBDS. Patients with deranged liver function tests (LFT), dilated CBD size more than 8 mm diameter, and history of pancreatitis are investigated by MRCP liberally. ERCP is reserved for therapeutic intentions on confirmation of CBDS. Patients with acute cholangitis and acute pancreatitis are managed according to the local algorithm [12,13]. We advocate a policy of universal cholecystectomy which incorporates index admission cholecystectomy for patients with mild to moderate acute cholangitis and/or acute biliary pancreatitis in addition to all suitable patients with acute cholecystitis as this not only reduces the length of stay and cost; but also has shown to restore the quality of life [13-15]. Selection criteria for LCBDE include imaging proven CBD stones, CBD size ≥8 mm, fitness for general anesthesia, and informed consent. LCBDE is advocated as an equally effective alternative to two-stage ERCP-LC, and all patients are given both treatment options and counseled accordingly.

Data collected include the patients’ biodata, underlying medical conditions, American Society of Anesthesiologists (ASA) status, clinical profile, hematological and radiological investigation results, and whether ERCP was performed before the surgery. Patients with the previous ERCP without duct clearance are also offered the option of LCBDE, and biliary stent (if present) is routinely removed during LCBDE. Operative details and perioperative outcomes are reported. No ethics committee approval was obtained as the data were extracted for audit from the surgeon’s operative case log, and no patient contact was made. We did not collect identifying patient details.

### 2.2. Operative technique

3D LCBDE was carried out under general anesthesia with 3D ENDOEYE FLEX^@^ (Olympus Medical Systems Corporation, Tokyo, Japan) flexible tip laparoscope system. The 3D system was compatible with 2D scopes, “plug and play,” “focus free,” and provides 100° range of movement in four directions while maintaining the correct image orientation. All patients received routine prophylactic antibiotics according to local antibiogram and international guidelines [16-18]. Patients were positioned supine with head elevation and right lateral elevated tilt. The four ports’ positions are similar to standard LC: infra-umbilical (10 mm), two right subcostals (5 mm), and one epigastric. We place 5 mm in epigastrium for standard LC, while for LCBDE, we prefer a 10 mm port for the insertion of 5 mm choledochoscope and ease of intracorporeal suturing of choledochotomy. Calot’s triangle was dissected to achieve a critical view of safety. The cystic artery was clipped and divided. The cystic duct was clipped in continuity to ensure no gallbladder stones drop into CBD. A longitudinal choledochotomy was performed using hook diathermy and scissors after adequate exposure of the supra-duodenal CBD. All the reported 3D LCBDE were done transcholedochal with 5 mm Olympus CHF Type V^@^ choledochoscope (Olympus Medical Systems Corporation, Tokyo, Japan) and primary repair without internal stent or T-tube. Choledochoscopy and Dormia basket^@^ (Boston Scientific Zero Tip Nitinol Stone Retrieval Basket, United States) extraction of calculi were performed until complete clearance of stones. CBD was inspected proximally up to second-order hepatic ducts. Choledochoscope was passed distally through the ampulla into the duodenum to document stone clearance. In patients where a 5 mm choledochoscope did not pass through the ampulla, a closed tip of Dormia basket^@^ was attempted to pass through the ampulla. In patients where even Dormia basket^@^ tip was unable to be passed through the ampulla, stat 1 g intravenous Glucagon was administered, and repeat attempts were made. We do not do intraoperative cholangiogram routinely to establish ductal clearance and reserve it for patients where choledochoscope or Dormia basket^@^ was unable to pass beyond the ampulla into the duodenum. The choledochotomy was repaired primarily with intracorporeal absorbable barbed suture V-Loc^@^ (Medtronic, Covidien, Minneapolis, United States of America) closure device 3-0 or 4-0. A tongue of healthy omentum was routinely tagged over the choledochotomy repair site with the same suture strand. Placement and removal of a closed suction abdominal drain are up to the surgeons’ preference. Following LCBDE, standard cholecystectomy was completed, and specimen with stones bagged into an impermeable disposable bag and extracted from the sub-umbilical incision. Port sites were infiltrated with 40 mls of 0.25% diluted bupivacaine.

Liver function tests were done for all patients postoperatively before discharge. According to the International Study Group of Liver Surgery definition, the biliary leak was defined, and its severity was graded [19]. The length of hospital stay was calculated from the date of admission to the date of discharge. The diagnosis of retained stone was opportunistic. Patients were not routinely subjected to imaging unless they deviate from ordinary post-operative courses, for example, bile leak or deranged liver enzymes. Retained stone was diagnosed when imaging showed residual CBDS following LCBDE. All patients had a minimum follow-up of 6 months. We summarized patient characteristics and study results using proportions and means or medians with minimum and maximum ranges using the Microsoft Excel software.

## 3. Results

Table 1 shows patient demography and pre-operative clinical profile. Twenty-seven consecutive patients with a mean age of 68 (range, 44–91) years underwent 3D LCBDE from July 2017 to January 2020. About 44% were male, and the median American Society of Anesthesiology (ASA) score was 2 (range 1–3). The most common comorbidity was hypertension (*n*=15, 56%). Indications for surgery were choledocholithiasis (*n*=18, 67%), cholangitis (*n*=6, 22%), and gallstone pancreatitis (*n*=3, 11%). Pre-operative MRCP was done in 74% (*n*=20) patients. In seven other patients, computerized tomography (CT) scan of the abdomen confirmed CBDS.

**Table 1:** Patient demography and preoperative clinical profile

	*n*=27 (%)
Mean age (years)	68 (range 44–91)
Gender	
Male	12 (44)
Female	15 (54)
American Society Anesthesiology score	
1	2 (7)
2	15 (56)
3	10 (37)
4	Nil
Comorbidity#	
Hypertension	15 (56)
Diabetes mellitus	3 (11)
Hyperlipidemia	11 (41)
Ischemic heart disease	5 (19)
Smoking (current active)	8 (30)
Body mass index (mean)	28.6 (range 18.5–34.5)
Clinical diagnosis	
Choledocholithiasis	18 (67)
Cholangitis	6 (22)
Acute biliary pancreatitis	3 (11)
Pre-operative haemoglobin (gm/dL)	12.4 (range 9.6–15.3)
Pre-operative Bilirubin (mol/L)	42.1 (range 9–215)
Preoperative Alkaline phosphatase (units)	198 (range 56–595)
Preoperative Imaging	
Computerized tomography scan	7 (26)
MRCP scan	20 (74)
Previous ERCP	
Yes	15 (56)
No	12 (44)
ERCP stenting	10

^#^Some patients had >1 comorbidity, MRCP – Magnetic resonance cholangiopancreatography, ERCP – Endoscopic retrograde cholangiopancreatography

Table 2 illustrates the operative details and postoperative outcome. CBD’s mean diameter was 14.5 mm (7–30 mm), and the mean stone size was 1.18 cm (range, 0.3–2.6 cm). Fifteen patients had pre-operative ERCP done. Among them, four patients had a failure to cannulated CBD, eight patients had a failure to extract CBDS, and three patients had ERCP with biliary stenting performed for cholangitis; no attempt was made to remove the stone. Median operative time was 160 (range 80–259) min, and all patients achieved CBD clearance intraoperatively on the choledochoscopy. About 68% (*n*=18) had an intra-operative drain inserted, and this was removed at a median of 4 (range 3–12) days. The median length of stay was 5 (range 2–20) days. Out of the 27 patients, one patient had the post-operative complication of bile leak, which settled with conservative management. A drain was kept and removed on POD 12. Another patient had retained stone that required an ERCP and stone removal despite achieving CBD clearance intraoperatively. This is suspected to be caused by dropped stones from the cystic duct after the choledochotomy suture repair. There was no open conversion, biliary stricture, 30-day readmission, or mortality at 6 months follow-up.

**Table 2:** Operative details and post-operative outcome

	*n*=27 (%)
Size of common bile duct (mm)(mean)(range)	14.5 (7–30)
Size of largest stone (cm)(mean)(range)	1.2 (0.3–2.6)
Number of stones	
Solitary	5 (19)
Multiple	22 (81)
Median operative time (minutes)(range)	160 (80–259)
Median blood loss (mls) (range)	30 (10–50)
Biliary stent removal (n=10)	10 (100)
Median length of stay (days)(range)	5 (2–20)
Drain inserted	
Yes	18 (67)
No	9 (33)
Median days to drain removal (range)	4 (3–12)
Histology#	
Acute on chronic cholecystitis	12 (44)
Chronic cholecystitis	14 (52)
Complications	
Bile leak	1 (4)
Retained stone	1 (4)
30-day readmission	Nil
Mortality at six months follow-up	Nil

^#^One patient did not have cholecystectomy done, and the only LCBDE performed

## 4. Discussion

Imaging technology and surgical innovation have impacted surgical advances. 3D laparoscopic camera systems are available since the first report in 1993 [20]. Despite more than two decades of existence, the adoption of 3D imaging in surgical laparoscopy is slow. In this report, we have shown that 3D LCBDE is safe and feasible. Furthermore, primary CBD repair with the use of absorbable barbed suture is safe.

Minimally invasive techniques replace open surgical techniques as first-line, and common bile duct exploration is no exception. Adoption of LC and ERCP has witnessed the pendulum swing away from open common bile duct exploration towards two-stage ERCP- LC in patients with CBDS [2-5,9]. The medical community embraced LC readily, but the adoption of advanced laparoscopic hepatobiliary procedures is slow [21,22]. This could be attributed to a lack of familiarity with intra-corporeal suturing, fear of uncontrollable bleeding, concerns about oncologic margins, less tactile feedback, and access to technology. In a prospective trial on 3D versus 2D laparoscopy, the surgical team reported a better definition of planes, precision, and depth perception with 3D laparoscopy. There is also less visual and muscle fatigue in 3D technology, especially in complicated and prolonged duration procedures [23]. None of the above studies include CBDE. In our experience, 3D imaging facilitates the Calot’s triangle dissection, choledochotomy, and intra-corporeal suturing of the bile duct with precision. Comparing the results of 3D LCBDE with our historical cohort of 18 patients managed by transcholedochal 2D LCBDE, median operative time was shorter (160 min (range 80–259 min) versus 250 min (range 160–415)) [22]. Although improved surgical experience could have reduced operating time, the previous patients were also operated upon by experienced surgeons, and the difference is not due to the learning curve effect. These results are similar to other published reports on the benefits of 3D laparoscopy in reducing operating time and shortening the learning curve. The benefit of an LCBDE with a 3D system is further proven by a recently published study by B Xiaobo comparing 2D versus 3D LCBDE. In this study, 3D LCBDE was found to have significantly shorter operative time, less blood loss, and less open conversion than the 2D group after propensity score matching. Despite our small series on 3D LCBDE, we noted a similar outcome in terms of hospital length of stay, operative duration, intraoperative blood loss, and post-operative complications compared to the study by B Xiaobo on 3D LCBDE. Of note, we have no open conversion as compared to an 8.5% conversion rate (18/213) in this study [24]. This could be due to the small sample size of our study. Implementing 3D LCBDE should be considered if resources permit, and all patients should be given a choice between two-stage ERCP-LC and single-stage LCBDE.

ERCP is associated with immediate risks such as bleeding, perforation, pancreatitis, and cholangitis [7]. Long-term sphincterotomy risks include recurrent ductal stones, stenosis of the papilla, and late development of cholangiocarcinoma, mainly a concern in younger patients [25]. LCBDE can avoid ERCP-associated risks. LCBDE is advantageous for the shorter hospital stay, fewer hospital visits, and lower costs [26-28]. Despite the above, one stage LCBDE is still less commonly practiced compared to ERCP + LC. In a study including 1961 patients with CBDS, one stage LCBDE was performed in only 28% of patients [29]. This is consistent with our local practice. The main factors that limit the utility of LCBDE are the lack of centralization of patients with biliary pathologies, lack of awareness among other disciplines, “culture and tradition” of ERCP, which is deeply rooted and, in some instances, ERCP is indeed mandatory to control acute sepsis. Our series reports all 3D LCBDE with a transcholedochal approach. This is relevant in patients with multiple or large stones, and it is easier to introduce a 5mm choledochoscope directly by a choledochotomy than via a trans-cystic route. The transcholedochal approach is safe in our experience, and 3D laparoscopy helps with precise suturing of the bile duct without stricture or bile leak. We routinely close the choledochotomy primarily, and in our opinion, “T-tube” should be considered of historical interest in the context of elective biliary surgery. Studies have shown that primary closure is superior to T-tube drainage with faster post-operative recovery, lower risk of post-operative bile leak, shorter surgery time and hospital stay, and superior quality of life [30,31].

Further, there is no evidence to show that primary closure with an internal biliary stent is superior compared to primary closure without an internal stent. Primary repair should be done by absorbable suture to avoid a nidus for future biliary lithiasis, and we use V-Loc^@^ (Medtronic, Covidien, Minneapolis, United States of America) barbed 3-0 or 4-0 suture. In our experience, using this suture facilitates the closure of choledochotomy without the need for intracorporeal knot tying as the suture end “eye” facilitates the end lock. The V-Loc^@^ suture was initially described for use in tendon repair to decrease the need for knot tying and increase gripping strength. It causes minimal tissue damage and has excellent hemostatic properties. There is also no need for repeated suture tightening and traction after each stitch. Its usage is reported to be safe and feasible in laparoscopic pancreaticojejunostomy anastomosis [32,33]. We also routinely use barbed suture for reconstituting laparoscopic subtotal cholecystectomy with a low incidence of bile leak rates [34,35]. However, the safety for V-loc sutures in bile duct anastomosis or repair is still unclear. Fernandez *et al*. reported the utilization of V-Loc^@^ suture for primary choledochotomy repair for LCBDE in 50 consecutive LCBDE patients between July 2012 and July 2014. They reported no bile leak, intra-abdominal collection, or need for reintervention [36]. In addition to the primary choledochotomy repair with V-Loc, we also routinely tagged a tongue of healthy omentum over the choledochotomy repair site to buttress the repair. We had one bile leak (1/27 = 3/7%), which was resolved with conservative management.

This non-randomized single-center small study describes our initial experience with 3D LCBDE and establishes the safety and feasibility of 3D laparoscopy in LCBDE. The lack of a control group in our study precludes any comparison with conventional 2D laparoscopy. However, the benefit of binocular vision with depth perception at negligible additional cost justifies that 3D LCBDE should be attempted where LCBDE capabilities exist. Further, it is essential to promote one-stage LCBDE as an equivalent treatment option to the two-stage ERCP-LC process, the current universal “default,” as one-stage LCBDE is proven to be safe with comparable. To initiate and adopt one stage LCBDE with primary CBD repair, we recommend selecting patients with CBD ≥ 1cm and smaller, non-impacted stones. We recommend utilizing 3D laparoscopy to provide better depth perception for facilitating intracorporeal suturing and reducing the learning curve. Barbed sutures are user-friendly and safe in our experience. We also recommend the careful use of a flexible choledochoscope to avoid equipment wear and tear. In conclusion, 3D LCBDE with primary CBD repair using absorbable barbed suture is safe and feasible.
